# Clinical Characteristics, Management and Outcomes of Nonintensive Care Unit Candidemia: Subanalysis of the ECMM *Candida* III Multinational European Observational Cohort Study

**DOI:** 10.1093/ofid/ofag133

**Published:** 2026-03-26

**Authors:** Stella Wolfgruber, Carolina Garcia-Vidal, Sarah Sedik, Jon Salmanton-García, Sevtap Arikan-Akdagli, Jean-Pierre Gangneux, Riina Rautemaa-Richardson, Valentina Arsić-Arsenijević, Sonia Martín-Pérez, Julio Dávila-Valls, Nurettin Erben, Emin Halis Akalin, Lubos Drgona, Cornelia Lass-Flörl, Julio García-Rodríguez, Tihana Bicanic, Helena Hammarström, Petr Hamal, Jörg Steinmann, Eelco F J Meijer, Nina Khanna, Guillaume Desoubeaux, Thomas Longval, Uluhan Sili, Janina Trauth, Matteo Bassetti, P Lewis White, Avinash Aujayeb, Tadeja Matos, Deniz Akyol, Francois Danion, Katrien Lagrou, Benedict Rogers, Maite Ruiz, Alena Siváková, Malgorzata Mikulska, Michael Samarkos, Ola Blennow, Deborah E A Lockhart, Blandine Denis, Birgit Willinger, Karin van Dijk, Ulrike Scharmann, Anna L Goodman, Jens van Praet, Manjusha Narayanan, Cristina Toscano, Laura Loughlin, Nick Alexander de Jonge, Alba Ruiz-Gaitan, Fanny Lanternier, Juergen Prattes, Emmanuel Roilides, Alida Fe Talento, Aleksandra Barac, Matthias Egger, Maiken Cavling Arendrup, Philipp Koehler, Oliver A Cornely, Martin Hoenigl, ECMM Candida III study group, Murat Akova, Christoph Zurl, Igor Stoma, Maria Calbacho, Alpay Azap, Johanna Kessel, Zdenek Racil, Lucia Taramasso, Anna Maria Peri, Matjaž Jereb, Ozge Turhan, Nikolai Klimko, Mario Tumbarello

**Affiliations:** Division of Infectious Diseases, Department of Internal Medicine, Medical University of Graz, Auenbruggerplatz 15, 8036 Graz, Austria; Translational Medical Mycology Research Unit, ECMM Excellence Center for Medical Mycology, Medical University of Graz, Graz, Austria; Department of Infectious Diseases, Hospital Clínic de Barcelona, Barcelona, Spain; Division of Infectious Diseases, Department of Internal Medicine, Medical University of Graz, Auenbruggerplatz 15, 8036 Graz, Austria; Translational Medical Mycology Research Unit, ECMM Excellence Center for Medical Mycology, Medical University of Graz, Graz, Austria; Institute of Translational Research, Cologne Excellence Cluster on Cellular Stress Responses in Aging-Associated Diseases (CECAD), Faculty of Medicine and University Hospital Cologne, University of Cologne, Cologne, Germany; Department I of Internal Medicine, Faculty of Medicine and University Hospital Cologne, Excellence Center for Medical Mycology (ECMM), University of Cologne, Cologne, Germany; German Centre for Infection Research (DZIF), Partner Site Bonn-Cologne, Cologne, Germany; Department of Medical Microbiology, Hacettepe University Medical School, Ankara, Türkiye; Univ Rennes, CHU Rennes, INSERM, EHESP, IRSET (Institut de Recherche en Santé Environnement Travail)—UMR_S 1085, Centre National de Référence des Mycoses et Antifongiques LA-AspC Aspergilloses Chroniques, European Excellence Center for Medical Mycology (ECMM-EC), F-35000 Rennes, France; Mycology Reference Centre Manchester, ECMM Excellence Centre, Department of Infectious Diseases, Wythenshawe Hospital, Manchester University NHS Foundation Trust, Manchester, UK; Division of Evolution, Infection, and Genomics, Faculty of Biology, Medicine, and Health, The University of Manchester, Manchester, UK; Medical Mycology Reference Laboratory, Institute of Microbiology and Immunology, Faculty of Medicine, University of Belgrade, Belgrade, Serbia; State University of Novi Pazar, Novi Pazar, Serbia; Hospital Nuestra Señora de Sonsoles, Ávila, Spain; Hospital Nuestra Señora de Sonsoles, Ávila, Spain; Department of Infectious Disease and Clinical Microbiology, Faculty of Medicine, Eskisehir Osmangazi University, Eskisehir, Türkiye; Department of Infectious Diseases and Clinical Microbiology, Faculty of Medicine, Bursa Uludağ University, Bursa, Türkiye; Department of Oncohematology, Comenius University and National Cancer Institute, Bratislava, Slovakia; Institute of Hygiene and Medical Microbiology, European Confederation of Medical Mycology Excellence Center for Medical Mycology, Department of Hygiene, Medical Microbiology and Virology, Innsbruck Medical University, Innsbruck, Austria; Microbiology Department, La Paz University Hospital, Madrid, Spain; Institute of Infection and Immunity, St George's University of London, London, UK; Department of Infectious Diseases, Institute of Biomedicine, Sahlgrenska Academy, University of Gothenburg, Gothenburg, Sweden; Department of Microbiology, Faculty of Medicine and Dentistry, Palacký University Olomouc, Olomouc, Czech Republic; Institute of Clinical Microbiology, Infectious Diseases and Infection Control, Paracelsus Medical University, Nuremberg, Germany; Department of Medical Microbiology and Immunology, Canisius-Wilhelmina Hospital (CWZ)/Dicoon, Nijmegen, The Netherlands; Radboudumc-CWZ Center of Expertise for Mycology, Nijmegen, The Netherlands; Department of Medical Microbiology, Radboud University Medical Center, Nijmegen, The Netherlands; Division of Infectious Diseases and Hospital Epidemiology, University Hospital of Basel, Basel, Switzerland; Department of Parasitology-Mycology-Tropical Medicine, Centre Hospitalier Régional Universitaire de Tours, Tours, France; Centre Hospitalier de Versailles, Hématologie, Le Chesnay, France; Department of Infectious Diseases and Clinical Microbiology, School of Medicine, Marmara University, Istanbul, Türkiye; Section of Infectious Diseases, Department of Medicine II, Justus-Liebig-University Giessen, Giessen, Germany; Department of Health Sciences (DISSAL), University of Genoa, Genoa, Italy; Infectious Diseases Unit, IRCCS Ospedale Policlinico San Martino, Genoa, Italy; Microbiology Division, Public Health Wales, Cardiff, UK; Centre for Trials Research and Division of Infection and Immunity, Cardiff University, Cardiff, UK; Northumbria Healthcare NHS Foundation Trust, North Shields, UK; Institute of Microbiology and Immunology, Faculty of Medicine, University of Ljubljana, Ljubljana, Slovenia; Department of Infectious Diseases, Ege University,Izmir, Turkey; Department of Infectious Diseases, Centre Hospitalier Universitaire de Strasbourg, Université de Strasbourg, Strasbourg, France; Laboratory of Clinical Microbiology, Department of Microbiology, Immunology and Transplantation, KU Leuven, Leuven, Belgium; Department of Laboratory Medicine and National Reference Center for Mycosis, UZ Leuven, Leuven, Belgium; Department of Clinical Microbiology, University Hospitals of Leicester NHS Trust, Leicester, UK; Unit of Infectious Diseases and Microbiology, Institute of Biomedicine of Seville, University Hospital Virgen del Rocío, Seville, Spain; Centro de Investigación Biomédica en Red de Enfermedades Infecciosas, Madrid, Spain; Department of Microbiology, St Anne's Faculty Hospital and Faculty of Medicine, Masaryk University, Brno, Czech Republic; Department of Health Sciences (DISSAL), University of Genoa, Genoa, Italy; Infectious Diseases Unit, IRCCS Ospedale Policlinico San Martino, Genoa, Italy; First Department of Medicine, Laikon General Hospital, Medical School, National and Kapodistrian University of Athens, Athens, Greece; Department of Infectious Diseases, Karolinska University Hospital, Stockholm, Sweden; Department of Medical Microbiology, Aberdeen Royal Infirmary, Aberdeen, UK; Institute of Medical Sciences, School of Medicine Medical Sciences and Nutrition, University of Aberdeen, Aberdeen, UK; Department of Infectious Diseases, Hôpital Saint-Louis-Lariboisière, AP-HP, Paris, France; Division of Clinical Microbiology, Department of Laboratory Medicine, Comprehensive Center for Infection Medicine, Medical University of Vienna, Vienna, Austria; Department of Medical Microbiology and Infection Prevention, Amsterdam University Medical Center, Academic Medical Center, Amsterdam Infection and Immunity Institute, Amsterdam, The Netherlands; Institute of Medical Microbiology, University Hospital Essen, University of Duisburg-Essen, Essen, Germany; Department of Infection, Guy's and St Thomas’ NHS Foundation Trust, London, UK; Medical Research Council Clinical Trials Unit, University College London, London, UK; Department of Nephrology and Infectious Diseases, AZ Sint-Jan Brugge AV, Brugge, Belgium; Department of Microbiology and Virology, Newcastle upon Tyne Hospitals NHS Foundation Trust, Newcastle upon Tyne, UK; Laboratory of Clinical Microbiology and Molecular Biology, Department of Clinical Pathology, Centro Hospitalar de Lisboa Ocidental, Lisboa, Portugal; Belfast Health and Social Care Trust, Belfast, UK; Department of Hematology, Amsterdam University Medical Centers, Amsterdam, The Netherlands; Department of Microbiology, Universitary and Politechnic Hospital La Fe, Health Research Institute La Fe, Valencia, Spain; Paris Cité Université, Necker Hospital, Assistance Publique-Hôpitaux de Paris, Paris, France; Division of Infectious Diseases, Department of Internal Medicine, Medical University of Graz, Auenbruggerplatz 15, 8036 Graz, Austria; Translational Medical Mycology Research Unit, ECMM Excellence Center for Medical Mycology, Medical University of Graz, Graz, Austria; Department of Infectious Diseases, Hippokration General Hospital, Medical School, Aristotle University of Thessaloniki, Thessaloniki, Greece; Department of Clinical Microbiology, ECMM Excellence Center, Trinity College Dublin, Dublin, Ireland; Department of Microbiology, Children’s Health Ireland at Temple Street, Dublin, Ireland; Department of Microbiology, Royal College of Surgeons in Ireland, Dublin, Ireland; Clinic for Infectious and Tropical Diseases, University Clinical Center of Serbia, Belgrade, Serbia; Division of Infectious Diseases, Department of Internal Medicine, Medical University of Graz, Auenbruggerplatz 15, 8036 Graz, Austria; Translational Medical Mycology Research Unit, ECMM Excellence Center for Medical Mycology, Medical University of Graz, Graz, Austria; Unit of Mycology, Statens Serum Institut, Copenhagen, Denmark; Department of Clinical Microbiology, Rigshospitalet, Copenhagen, Denmark; Department of Clinical Medicine, University of Copenhagen, Copenhagen, Denmark; Institute of Translational Research, Cologne Excellence Cluster on Cellular Stress Responses in Aging-Associated Diseases (CECAD), Faculty of Medicine and University Hospital Cologne, University of Cologne, Cologne, Germany; Department I of Internal Medicine, Faculty of Medicine and University Hospital Cologne, Excellence Center for Medical Mycology (ECMM), University of Cologne, Cologne, Germany; Department I of Internal Medicine, Division of Clinical Immunology, Faculty of Medicine, University Hospital Cologne, University of Cologne, Cologne, Germany; Institute of Translational Research, Cologne Excellence Cluster on Cellular Stress Responses in Aging-Associated Diseases (CECAD), Faculty of Medicine and University Hospital Cologne, University of Cologne, Cologne, Germany; Department I of Internal Medicine, Faculty of Medicine and University Hospital Cologne, Excellence Center for Medical Mycology (ECMM), University of Cologne, Cologne, Germany; German Centre for Infection Research (DZIF), Partner Site Bonn-Cologne, Cologne, Germany; Faculty of Medicine and University Hospital Cologne, Clinical Trials Centre Cologne (ZKS Köln), University of Cologne, Cologne, Germany; Division of Infectious Diseases, Department of Internal Medicine, Medical University of Graz, Auenbruggerplatz 15, 8036 Graz, Austria; Translational Medical Mycology Research Unit, ECMM Excellence Center for Medical Mycology, Medical University of Graz, Graz, Austria; BioTechMed-Graz, Graz, Austria

**Keywords:** candidemia, disease outcome, invasive fungal infections, non-ICU

## Abstract

The European Confederation of Medical Mycology *Candida* III was a pan-European, multicenter observational study of adult patients with blood culture–proven candidemia. Among a total of 632 patients with candidemia across 64 institutions in 20 European countries, a subanalysis of 396 (63%) cases occurring outside the intensive care unit (ICU) was conducted. Compared with ICU patients, non-ICU patients had a higher comorbidity burden (median Charlson comorbidity index [CCI] 6 vs 5 in ICU patients, *P* = .006). Hematologic and oncologic malignancies were more frequent among non-ICU cases (45.5% vs 28.4%, *P* < .001), whereas both chronic kidney and cardiovascular disease were more prevalent in ICU patients (*P* < .001). Non-ICU patients had significantly lower mortality in Kaplan–Meier survival analysis (*P* > .001). Postsurgical non-ICU patients (n = 45) had the highest survival rate (73.3%, *P* = .003) and the longest hospital stay, even after excluding all cases with a fatal outcome before day 30. In non-ICU patients, older age, hemato-oncologic malignancies, chronic liver disease, and COVID-19 were all independently associated with mortality risk, while treatment consultation by an infectious disease or clinical microbiology consultant, and initial treatment with an echinocandin, respectively, higher EQUAL *Candida* scores were associated with lower mortality risk in the multivariable Cox regression models. In conclusion, despite higher comorbidity rates, non-ICU patients with candidemia had higher survival rates.

## Teaser

A subanalysis of 396 candidemia cases diagnosed outside the intensive care unit from the pan-European multicenter ECMM *Candida* III study showed higher survival rates despite greater comorbidity. Increased mortality was associated with older age, hematological malignancies, chronic liver disease, and severe acute respiratory syndrome coronavirus 2 infection, whereas infectious disease or clinical microbiology consultation, initial echinocandin therapy, and higher EQUAL *Candida* scores were linked to improved survival.

Invasive candidiasis (IC) and candidemia remain the most common invasive fungal diseases in many parts of the world [1–3]. Candidemia accounts for ∼626 000 cases worldwide each year and is associated with high morbidity and mortality [4, 5]. In Europe, the all-cause 90-day mortality rate among adults with candidemia remains above 40% [6].

*Candida albicans* remains the most frequently isolated species in cases of candidemia and IC. However, over the past decades, there has been a global shift toward non-*albicans Candida* species, such as *Nakaseomyces glabratus* (formerly known as *Candida glabrata*), *Candida tropicalis*, *Candida parapsilosis*, as well as the emerging multidrug-resistant *Candida auris* (also referred to as *Candidozyma auris*) [5, 7–9]. Across Europe, significant differences in species distribution are observed. In Northern Europe, *C. glabrata* was the second most common isolated species, whereas *C. parapsilosis* was less common. In contrast, in Southern and Mediterranean countries (eg, Italy, Spain, and Greece), *C. parapsilosis* and, in some countries, *C. auris* have been predominant among *non-albicans Candida* spp., while *C. glabrata* was less frequent [10]. Species distribution and antifungal resistance patterns also differ between ICU and non-ICU settings, supporting separate subgroup analysis by care setting [11].

Over the past 25 years, the European Confederation of Medical Mycology (ECMM) has conducted 3 large-scale, multicenter cohort studies (ECMM *Candida* I, II, and III) to investigate the epidemiology, risk factors, and outcomes associated with IC in Europe [6, 12, 13].

Throughout these studies, ICU admission has remained one of the most important risk factors for candidemia [2], and ICU-associated candidemia has been the subject of numerous studies in recent decades [14, 15]. In contrast, candidemia occurring outside the ICU has received less attention, with larger investigations focusing on this subgroup being rare [16, 17]. In this subanalysis of the ECMM *Candida* III study, the primary objective was to compare the clinical characteristics, management, and outcomes of candidemia in non-ICU versus ICU settings. As a secondary objective, we aimed to explore heterogeneity within the non-ICU population by stratifying patients into 3 subgroups: patients with hematologic or oncologic malignancies, postsurgical patients, and other non-ICU patients not included in the first 2 categories.

## METHODS

### Study Design

For the ECMM *Candida* III study, data were collected from 64 institutions across 20 countries in Europe [2, 6–8, 10, 18]. During the study period from 1 July 2018 to 31 March 2022, each participating hospital included the first 10 consecutive adult patients diagnosed with candidemia. Non-ICU patients were defined as those not located in an ICU at the time the first positive blood culture was drawn. Patients transferred to the ICU after the blood culture was drawn were classified according to their location at the time of first blood culture collection and were not reclassified. Malnutrition was assessed solely based on body mass index (BMI). Underweight was defined as a BMI < 18.5 kg/m^2^, in accordance with standard WHO definitions. Patient data were systematically recorded using an electronic case report form integrated into the ECMM Candida Registry (FungiScope, CandiReg; NCT01731353) [19], resulting in a total cohort of 632 patients. The EQUAL *Candida* score, developed by the ECMM in 2018, was used to evaluate the quality of candidemia management and measure adherence to key diagnostic and therapeutic recommendations from the 2 major American and European clinical guidelines [6, 20, 21]. Scores were normalized according to the presence or absence of a central venous catheter (CVC). The Ostrosky-Zeichner score was calculated as follows: a patient was considered positive if any systemic antibacterial agent was administered or a CVC was present and at least 2 of the following factors were present: total parenteral nutrition, dialysis, major surgery, use of steroids, or use of other immunosuppressive agents [22]. The study complied with the Declaration of Helsinki. The University of Cologne (EK 17-485) granted central ethical approval, in addition, local confirmation or approval was required in accordance with local regulations.

This subanalysis used the full dataset from the parent study [6], focusing specifically on patients treated outside the ICU. We investigated risk factors, underlying diseases, causative species, treatment, and clinical outcomes, comparing the results with those of the ICU population. For this purpose, the complete dataset was filtered to include non-ICU patients only. These cases were then categorized into 3 groups: patients with hematologic malignancies or solid tumors, patients who had undergone major surgery and the remaining patients who could not be categorized into the other groups. Patients in the hemato-oncologic malignancies group included those with a hematologic malignancy or a solid tumor, regardless of whether they had undergone major surgery. Patients who had undergone major surgery but did not have a malignancy were assigned to the postsurgery group. This group included individuals who had abdominal or visceral surgery, urologic, or gynecologic surgery, cardiothoracic, neurosurgical, orthopedic, or trauma surgery, head and neck surgery, wound surgery, as well as major endoscopic or interventional procedures.

### Statistical Analysis

Statistical analyses were performed using IBM SPSS Statistics for Windows, version 28 (IBM Corp., Armonk, NY) and SAS 9.4 (2002–2016 by SAS Institute Inc., Cary, NC).

Descriptive statistics were used to summarize the data, presenting categorical variables as counts and percentages and continuous variables as median with interquartile range (IQR). Group comparisons for categorical variables were performed using Pearson χ^2^ test with Fisher's exact test applied when the χ^2^ requirements were not met. Continuous variables were compared using the Mann–Whitney *U* test. For comparisons among the 3 non-ICU subgroups categorical variables were analyzed using the χ^2^ test or Fisher's exact test, while continuous variables were compared using the Kruskal–Wallis test.

The day of diagnosis was defined as the day when the first positive blood culture results for *Candida* spp. became available to the treating physician. For time-specific mortality analyses at 14, 30, 90, and 180 days from the day of diagnosis, only patients with documented vital status at the respective time point were included. Patients who were alive at the last day of follow-up but had no documented follow-up beyond the respective time points were excluded from the denominator.

For the Cox regression analyses, the patients were followed until they died or had their last documented date of study follow-up. They were censored on the date of their last follow-up if they had not died.

Variables were separately analyzed using univariable Cox proportional hazard models. Hazard ratios (HRs) and 95% confidence intervals (CIs) were calculated. Assumptions for the models were checked, and in case of nonproportional hazards, time-dependent covariates were included in the model. Variables showing a univariate *P*-value of <.2 were considered for further analysis. Within the set of significant predictors, multicollinearity was analyzed and parameters were excluded if multicollinearity or reverse causation bias was evident. The resulting variables were analyzed using a backwards strategy to obtain a parsimonious model.

A *P*-value of <.05 was considered statistically significant.

## RESULTS

The entire study cohort included 632 patients from 20 European countries (Supplementary Figure 1 and Supplementary Table 1) with blood culture–proven candidemia. Of these patients, 396 (62.7%) were not in the ICU at the time of diagnosis, and there was no missing data regarding ICU admission status.

Table 1 displays patient demographics, underlying diseases, and risk factors for candidemia among non-ICU versus ICU patients. Of 396 patients diagnosed with candidemia outside the ICU, 88 (22.2%) were admitted to the ICU after diagnosis of candidemia. There were no significant differences in sex distribution or age between ICU and non-ICU patients. Hemato-oncologic malignancies were significantly more prevalent in non-ICU patients than in ICU patients (*P* < .001). Conversely, chronic cardiovascular disease (*P* < .001), acute or chronic renal disease (*P* < .001), diabetes mellitus (*P* = .002), rheumatoid diseases (*P* = .037), and COVID-19 infection (*P* < .001) were less prevalent in non-ICU patients. Major surgery was more common among ICU patients compared with non-ICU patients (*P* < .001). Low serum albumin levels were observed more frequently in ICU patients versus non-ICU patients (*P* < .001). Obesity (BMI > 30) was more prevalent in ICU patients than in non-ICU patients (*P* < .001). Underweighted individuals (BMI < 18.5) were more prevalent in the non-ICU cohort (*P* < .001).

**Table 1.** Patient Demographics, Underlying Diseases, and Risk Factors for Candidemia

	Study Cohort (n = 632)	Non-ICU (n = 396)	ICU (n = 236)	*P*-Value
Sex				.505
Male	369 (58.4%)	227 (57.3%)	142 (60.2%)	
Female	263 (41.6%)	169 (42.7%)	94 (39.8%)	
Age at candidemia diagnosis				.400
18–29 y	17 (2.7%)	13 (3.3%)	4 (1.7%)	
30–49 y	101 (16.0%)	65 (16.4%)	36 (15.3%)	
50–69 y	299 (47.3%)	180 (45.5%)	119 (50.4%)	
70–89 y	197 (31,2%)	124 (31.3%)	73 (30.9%)	
>90 y	18 (2.8%)	14 (3.5%)	4 (1.7%)	
Underlying diseases				
Hemato-oncologic malignancy	248	181 (45.7%)	67 (28.4%)	<.001
Chronic cardiovascular disease	149	58 (14.6%)	91 (38.6%)	<.001
Chronic liver disease	60	35 (8.6%)	25 (11.0%)	.466
Chronic pulmonary disease	62	32 (8.1%)	30 (12.7%)	.072
Acute or chronic renal disease	137	64 (16.2%)	73 (30.9%)	<.001
Diabetes mellitus	139	72 (18.2%)	67 (28.4%)	.002
Rheumatoid disease/autoimmune disorder	32	14 (3.5%)	18 (7.6%)	.037
HIV/AIDS	10	7 (1.8%)	3 (1.3%)	.751
COVID-19	26	1 (0.3%)	25 (10.6%)	<.001
Risk factors for candidemia				
Solid organ transplantation	15	8 (2.3%)	7 (2.5%)	.450
Other disorders requiring or causing immunosuppression	27	16 (4.0%)	11 (4.7%)	.690
Charlson comorbidity index, median (IQR)	5 (3–8)	6 (3–8)	5 (3–7)	.006
Major surgery (not including surgery as antifungal therapy)	165	77 (19.4%)	88 (37.3%)	<.001
Other risk factors (eg, prosthetic material, prosthetic valve/foreign body)	78	45 (11.4%)	33 (14.0%)	.382
Trauma	24	12 (3.0%)	12 (5.1%)	.202
Alcoholism	44	27 (6.8%)	17 (7.2%)	.872
Burn	6	2 (0.5%)	4 (1.7%)	.203
IV drug abuse	16	12 (3.0%)	4 (1.7%)	.434
Low albumin level	251	123 (31.1%)	128 (54.2%)	<.001
Obesity (BMI > 30)^*a*^	69	27 (6.8%)	42 (17.8%)	<.001
Underweight (BMI < 18.5)^*a*^	33	28 (6.6%)	5 (2.1%)	.009
CVC	399	192 (48.5%)	207 (87.7%)	<.001
Positive Ostrosky-Zeichner score	224	NA	30/224 (13.4%)	NA

Percentages are calculated using the total number of patients in each group. Patients may have >1 underlying disease or risk factor, so counts in these categories can overlap.

Abbreviations: BMI, body mass index; COVID-19, coronavirus disease 2019; CVC, central venous catheter; IQR, interquartile range.

^*a*^BMI data unavailable for 181 non-ICU patients (45.7%) and 98 ICU patients (41.5%).

The median Charlson comorbidity index (CCI) was higher in non-ICU patients than in ICU patients (median 6, IQR 3–8 vs median 5, IQR 3–7; *P* = .006).

Causative *Candida* species are displayed in Table 2. Species distribution by participating country is provided in Supplementary Table 2. *Candida albicans* was the predominant causative species isolated in 44.2% of non-ICU patients. This was followed by *N. glabratus*, identified in 22% of non-ICU patients. No difference was observed between the groups for either species. However, a significant group-specific distribution was observed for *Pichia kudriavzevii* (formerly known as *Candida krusei*) which was predominantly isolated in the non-ICU group (3.5% vs 0.8% in ICU patients; *P* = .038). In patients with *P. kudriavzevii* candidemia, the 30-day mortality rate was significantly higher in the non-ICU cohort (66.7%) compared with ICU patients (33.3%, *P* = .035), a finding that was not attributable to inadequate first-line therapy as all patients with fatal outcomes received echinocandins as first-line therapy. *Candida auris* was significantly more prevalent in the ICU cohort (5.9%) than in non-ICU patients (0.3%; *P* = <.001). Similarly, *Meyerozyma guilliermondii* (formerly known as *Candida guilliermondii*) was only found in the ICU group (2.1%), whereas *Clavispora lusitaniae* (formerly known as *Candida lusitaniae*) only in non-ICU patients (1.5%), although the numbers of cases were small and the differences not statistically significant (Table 2).

**Table 2.** Consultation, Diagnostics, Management, Outcome, and Causative Species in Non-ICU Versus ICU Patients

	Study Cohort (n = 632)	Non-ICU (n = 396)	ICU (n = 236)	*P*-Value
Consultation				
Infectious diseases consultation	317	203 (51.3%)	114 (48.3%)	.511
Microbiology consultation	185	115 (29.0%)	70 (29.7%)	.928
No consultation	118	76 (19.2%)	42 (17.8%)	.752
Diagnostics				
Initial blood cultures (≥40 mL)	557	346 (87.4%)	211 (89.4%)	.525
Species identification	570	352 (88.9%)	218 (92.4%)	.169
Susceptibility testing	520	333 (84.1%)	187 (79.2%)	.197
Echocardiography	267	174 (43.9%)	93 (39.4%)	.280
Ophthalmoscopy	219	162 (40.9%)	57 (24.2%)	<.001
Management				
Initial echinocandin treatment	353	213 (53.8%)	140 (59.3%)	.186
Stepdown to fluconazole	186	133 (33.6%)	53 (22.5%)	.003
Treatment for ≥14 d after the first negative blood culture	325	208 (52.5%)	117 (49.6%)	.511
CVC removal ≤24 h from diagnosis^*a*^	216	112/192 (58.3%)	104/207 (50.2%)	.109
CVC removal >24 but <72 h from diagnosis^*a*^	82	49/80 (61.3%)	33/103 (32.0%)	<.001
EQUAL *Candida* scores relative to max (median, IQR)	0.68 (0.50–0.84)	0.68 (0.50–0.86)	0.68 (0.49–0.82)	.187
Outcome^*b*^				
Total duration of hospitalization after day 0, days, median (IQR)	15 (4–30)	15 (5–30)	15 (2–33)	.987
Total duration of hospitalization for survivors at day 30, median (IQR)	21 (10–43)	21 (10–35)	27 (14–49)	.016
Ocular involvement	19	13 (3.3%)	6 (2.5%)	.397
Cardiac involvement	25	15 (3.8%)	10 (4.2%)	.664
Mortality				
14-d mortality	166/556	93/342 (27.2%)	73/214 (34.1%)	.083
30-d mortality	212/498	119/304 (39.1%)	93/194 (47.9%)	.053
90-d mortality	251/415	140/246 (56.9%)	111/169 (65.7%)	.072
180-d mortality	261/354	144/198 (72.7%)	117/156 (75%)	.629
Patient alive at last follow-up	331	233/386 (60.4%)	98/230 (42.6%)	<.001
*Candida* species				
*C. albicans*	287	175 (44.2%)	112 (47.5%)	.458
*Nakaseomyces glabratus (C. glabrata)*	133	87 (22.0%)	46 (19.5%)	.482
*C. parapsilosis*	83	52 (13.1%)	31 (13.1%)	1.000
*C. tropicalis*	46	33 (8.3%)	13 (5.5%)	.208
*Pichia kudriavzevii* (*C. krusei*)	16	14 (3.5%)	2 (0.8%)	.038
*Candidozyma auris* (*C. auris*)	15	1 (0.3%)	14 (5.9%)	<.001
*C. dubliniensis*	10	4 (1.0%)	6 (2.5%)	.187
*Clavispora lusitaniae* (*C. lusitaniae*)	6	6 (1.5%)	0	.089
*Kluyveromyces marxianus* (*C. kefyr*)	5	3 (0.8%)	2 (0.8%)	1.000
*Meyerozyma guilliermondii* (*C. guilliermondii*)	6	0	6 (2.5%)	.003
Others^*c*^	13	12 (3.0%)	1 (0.4%)	.061

Unless otherwise indicated, percentages are calculated based on all patients in the non-ICU and ICU subgroups.

Abbreviations: CVC, central venous catheter; IQR, interquartile range.

^*a*^For the removal of CVC, the percentages are based on the number of patients who still had a CVC at the respective time point.

^*b*^Percentages have been calculated based on the number of survivors with follow-up data beyond each of the respective time points.

^*c*^Others: *Debaryomyces hansenii* (*C. famata*): non-ICU n = 1, ICU n = 1; *Pichia cactophila* (*C. inconspicua*): non-ICU n = 2, ICU n = 0; *C. norvegensis*: non-ICU n = 1, ICU n = 0; *Wickerhamomyces anomalus* (*C. pelliculosa*): non-ICU n = 2, ICU n = 0; *Diutina rugosa* (*C. rugosa*): non-ICU n = 3, ICU n = 0; *C. digboensis*: non-ICU n = 1, ICU n = 0; *C. orthopsilosis*: non-ICU n = 1, ICU n = 0; *Saccharomyces cerevisiae*: non-ICU n = 1, ICU n = 0; species unidentified: non-ICU = 1, ICU = 5. Data on *Candida* species was missing from 10 patients. In mixed infections, each isolated species was counted separately, so that all isolates in a sample were included in the respective species-specific numbers.

Details on management and outcomes of candidemia are shown in Table 2. Antifungal susceptibility testing was performed on isolates from 64% of non-ICU patients and 79.2% of ICU patients. Ophthalmoscopy was performed more frequently in non-ICU patients (*P* < .001). Central venous catheter removal within 24 hours of diagnosis was observed in 58.3% of non-ICU cases that were not admitted to the ICU, compared with 50.2% of ICU patients (*P* = .109). Delayed CVC removal between 24 and 72 hours after diagnosis was more common in non-ICU patients (61.2% vs 32.0% of those in whom CVC was not removed within 24 hours, *P* < .001). An echinocandin, as recommended in current guidelines, was used as the initial therapy in 53.8% of non-ICU patients and 59.3% of ICU patients (*P* = .186). Stepdown to fluconazole occurred significantly more often in the non-ICU group than in the ICU group (33.4% vs 22.8%; *P* = .005). Among patients who survived ≥14 days after the diagnosis (n = 442), stepdown therapy was performed in 41.0% of non-ICU and 29.2% of ICU patients (*P* = .017).

The median duration of hospitalization among those who survived at least 30 days after diagnosis (Table 2) was significantly shorter in non-ICU patients versus ICU patients (21 days, IQR 10–35 days vs 27 days, IQR 14–49 days, *P* = .016).

EQUAL *Candida* scores, normalized to the maximum possible score (19 for those without CVC and 22 for those with CVC) were comparable between the groups (Table 2). Ostrosky-Zeichner score positivity was met by 13.4% (30/224) of ICU patients.

**Figure 1.**
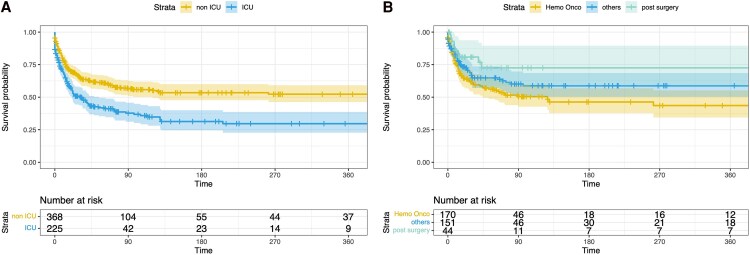
*A*, Kaplan–Meier survival curve showing the probability of survival of nonintensive care unit (ICU) (yellow) and ICU patients (blue). *P* < .001. *B*, Kaplan–Meier survival curve showing the probability of survival in non-ICU patients. Groups: HemOnc malignancy (yellow), post major surgery (green), others (blue). *P* = .039 (log rank test).

Mortality tended to be consistently lower in the non-ICU cohort at all time points except day 180 (Table 2). Non-ICU patients also had significantly higher survival rates at the last follow-up compared with ICU patients (60.4% vs 42.6%; *P* < .001). Kaplan–Meier survival analysis (Figure 1*A*) confirmed a significantly lower survival probability (*P* < .001) in the ICU group.

Results of the Cox regression analysis (Supplementary Table 3) showed that treatment in the ICU at the time of candidemia diagnosis was associated with a significantly increased mortality risk in univariable analysis (HR 1.76, 95% CI 1.39–2.23, *P* < .001), and remained an independent predictor in multivariable analysis (adjusted HR 1.90, 95% CI 1.49–2.41, *P* < .001), together with higher CCI (adjusted HR 1.10, 95% CI 1.06–1.14, *P* < .001).

### Subanalysis of the Nonintensive Care Unit Cohort

To further analyze the non-ICU cohort (n = 396), patients were stratified into 3 subgroups: patients with hemato-oncologic malignancies (n = 181), those with major surgery (post major surgery, n = 45), and all other patients (others, n = 170; Table 3).

**Table 3.** Subanalysis of Non-ICU Patients

	Non-ICU Cohort (n = 396)	Hemato-oncologic Malignancies (n = 181)	Post Major Surgery (n = 45)	Others (n = 170)	*P*-Value
Sex					
Male	227	100 (55.2%)	22 (48.9%)	105 (51.1%)	.223
Female	167	81 (44.8%)	23 (51.1%)	65 (38.2%)	
Causative species					
*C. albicans*	173	80 (44.2%)	14 (31.1%)	79 (46.5%)	.178
*N. glabratus*	85	36 (19.9%)	12 (26.7%)	37 (21.8%)	.607
*C. parapsilosis*	51	21 (11.6%)	6 (13.3%)	24 (14.1%)	.777
*C. tropicalis*	33	21 (11.6%)	2 (4.4%)	10 (5.9%)	.093
*C. auris*	1	0	1 (2.2%)	0	-
*P. kudriavzevii*	14	9 (5.0%)	0	5 (2.9%)	.232
*C. dubliniensis*	4	1 (0.6%)	1 (2.2%)	2 (1.2%)	.581
*Cl. lusitaniae*	6	2 (1.3%)	1 (1.3%)	3 (1.8%)	.940
*K. marxianus*	3	2 (1.1%)	0	1 (0.6%)	.705
Risk factors					
Central venous catheter	192	104 (57.5%)	24 (53.3%)	64 (37.6%)	<.001
Low albumin level	124	67 (37.0%)	10 (22.2%)	47 (27.6%)	.063
Obesity (BMI > 30)	27	15 (12.3%)	2 (11.8%)	10 (12.7%)	.994
Underweight (BMI < 18.5)	28	18 (14.9%)	1 (5.9%)	9 (11.4%)	.516
Diabetes mellitus	72	26 (14.4%)	9 (20.0%)	37 (21.8%)	.188
Chronic liver disease	35	7 (3.9%)	5 (11.1%)	23 (13.5%)	.005
Solid organ transplantation	8	0	1 (2.2%)	7 (4.1%)	.23
Charlson comorbidity index	6 (3–8)	6 (4–9)	5 (3–8)	5 (3–8)	.041
Consultation					
Infectious diseases consultant	203	99 (54.7%)	23 (51.1%)	81 (47.6%)	.870
Microbiology consultant	115	39 (21.5%)	14 (31.1%)	62 (36.5%)	.820
No consultation	76	42 (23.3%)	8 (17.8%)	26 (15.3%)	.835
Diagnostics					
Initial blood cultures (40 mL)	346	171 (94.5%)	32 (71.1%)	143 (84.1%)	<.001
Species identification	352	170 (93.9%)	35 (77.8%)	147 (86.5%)	.004
Susceptibility testing	333	163 (90.1%)	33 (73.3%)	137 (80.6%)	.006
Echocardiography	174	75 (41.4%)	17 (37.8%)	82 (48.2%)	.297
Ophthalmoscopy	162	70 (38.7%)	20 (44.4%)	72 (42.4%)	.686
Management					
Initial echinocandin treatment	213	100 (55.2%)	19 (42.2%)	94 (55.3%)	.255
Stepdown to fluconazole	133	64 (35.4%)	12 (26.7%)	57 (33.5%)	.543
Treatment for ≥14 d after the first negative blood culture	208	89 (49.2%)	23 (51.1%)	96 (56.5%)	.384
CVC removal ≤24 h from diagnosis^*a*^	112	53/104 (51.0%)	18/24 (75.0%)	41/64 (64.1%)	.101
CVC removal >24 <72 h from diagnosis^*a*^	49	26/51 (51.0%)	5/6 (83.3%)	18/23 (78.3%)	.541
EQUAL *Candida* scores relative to max (median, IQR)	0.68 (0.50–0.86)	0.68 (0.50–0.84)	0.64 (0.29–0.86)	0.73 (0.45–0.86)	.635
Outcome					
Total duration of Hospitalization after day 0, days, median (IQR)	15 (5–30)	13 (5–29)	17 (9–45)	15 (2–27)	.035
Total duration of hospitalization for survivors at day 30, median (IQR)	21 (10–35)	21 (10–36)	32 (14–53)	20 (7–31)	.037
Ocular involvement	13	8 (14.0%)	1 (6.3%)	4 (7.3%)	.426
Cardiac involvement	15	5 (8.1%)	1 (7.1%)	9 (13.2%)	.575
Mortality^*b*^					
14-d mortality	93	49/158 (31%)	8/41 (19.5%)	36/143 (25.2%)	.262
30-d mortality	119	62/147 (42.2%)	8/34 (23.5%)	49/123 (39.8%)	.131
90-d mortality	140	75/122 (61.5%)	10/23 (43.5%)	55/101 (54.5%)	.226
180-d mortality	144	78/96 (81.3%)	10/17 (58.8%)	56/85 (65.9%)	.028
Patient alive at last follow-up	233/383	98/179 (54.7%)	33/45 (73.3%)	102/159 (64.2%)	.003

Abbreviations: BMI, body mass index; CVC, central venous catheter; ICU, intensive care unit; IQR, interquartile range.

^*a*^For the removal of CVC, the percentages are based on the number of patients who still had a CVC at the respective time point.

^*b*^Percentages have been calculated based on the number of survivors with follow-up data beyond each of the respective time points.

While there was no significant difference in *Candida* spp. distribution across the 3 subgroups, *C. tropicalis* tended to be detected more frequently in patients with hemato-oncologic malignancies (*P* = .093).

Central venous catheter presence was more frequent in the hemato-oncologic malignancies (57.5%) and post major surgery groups (53.3%) compared with others (37.6%; *P* < .001). The hemato-oncologic malignancy group had a higher CCI (*P* = .041), while median hospital stay (in those who survived at least 30 days) was longest in the postsurgery group (median 32 days IQR 14–53 days), compared with hemato-oncologic malignancy patients (median 21 days, IQR 10–36 days) and other patients (median 20 days, IQR 7–30 days, *P* = .037). In a small subgroup of 11 patients (5 hemato-oncologic malignancies, 2 post major surgery, and 4 others), no antifungal therapy was reported, despite survival for at least 2 days after diagnosis. All these patients ultimately died, suggesting a potential palliative treatment setting in this subgroup. Survival rates at the last follow-up were highest in postsurgery patients (73.3%), followed by others (64.2%) and hemato-oncologic malignancy patients (54.7%; *P* = .003). Thirty-day mortality was 42.2% in the hemato-oncologic malignancy group, 23.5% in the post major surgery group, and 39.8% in the others group (*P* = .132).

Figure 1 *B* shows Kaplan–Meier survival curves for the 3 different subgroups of non-ICU patients. Patients with hemato-oncologic malignancies had the lowest survival probability (*P* = .039).

Univariable Cox regression analysis of non-ICU patients (Table 4) showed that CCI was significantly associated with increased mortality risk (HR per point increase 1.11, 95% CI 1.06–1.16, *P* < .001), as were chronic liver disease (HR 1.85, 95% CI 1.14–3.00, *P* = .013) and hemato-oncologic malignancies (HR 1.45, 95% CI 1.05–2.00, *P* = .025), whereas major surgery was associated with lower mortality risk (HR 0.60, 95% CI 0.38–0.95, *P* = .029). Treatment consultations by an infectious disease consultant or microbiology consultant were associated with significantly lower risk for mortality (HR 0.49, 95% CI .34–.71, *P* < .001), as were multiple individual variables of the EQUAL *Candida* scores, such as echocardiography, ophthalmoscopy, and initial treatment with an echinocandin (all *P* < .001), and, consequently, also higher EQUAL *Candida* scores (HR 0.14 per percent increase relative to max, 95% CI .08–.23, *P* < .001).

**Table 4.** Univariable and Multivariable Cox Regression Model for Predictors of Mortality for Non-ICU Patients

	Univariable HR (95% CI)	*P*-Value
Sex (male vs female)	0.81 (0.58–1.12)	.196
Age at candidemia diagnosis (per year)	1.02 (1.01–1.03)	<.001
Underlying diseases		
Hemato-oncologic malignancies	1.45 (1.05–2.00)	.025
Rheumatic disease/autoimmune disorder	1.51 (0.74–3.08)	.259
Chronic cardiovascular disease	1.12 (0.73–1.74)	.599
Chronic liver disease	1.85 (1.14–3.00)	.013
Solid organ transplantation	0.47 (0.12–1.88)	.283
Acute or chronic renal disease	1.10 (0.72–1.68)	.668
Diabetes mellitus	0.72 (0.46–1.12)	.147
COVID-19	6.03 (0.81–45.05)	.169
Risk factors for candidemia		
Charlson comorbidity index	1.11 (1.06–1.16)	<.001
Major surgery (not including surgery as antifungal therapy)	0.60 (0.38–0.95)	.029
Trauma	1.45 (0.68–3.10)	0.365
Alcoholism	1.50 (0.85–2.65)	.189
Low albumin level	1.27 (0.91–1.77)	.172
Central venous catheter	1.11 (0.80–1.53)	.539
Diagnostics and management		
EQUAL *Candida* score relative to max (per %)	0.14 (0.08–0.23)	<.001
Initial blood cultures (>40 mL)	0.71 (0.44–1.17)	.197
Echocardiography performed	0.39 (0.27–0.55)	<.001
Ophthalmoscopy performed	0.31 (0.22–0.45)	<.001
Start with an echinocandin	0.53 (0.38–0.73)	<.001
Stepdown to fluconazole	0.38 (0.26–0.57)	<.001
Treatment consultation by an infectious diseases or microbiology consultant	0.49 (0.34–0.71)	<.001
Model 1	Multivariable adjusted HR (95% CI)	*P*-value
Treatment consultation by an infectious diseases or microbiology consultant	0.38 (0.21–0.69)	.001
Chronic liver disease	3.47 (1.51–7.98)	.003
Age (per year)	1.02 (1.00–1.04)	.032
EQUAL *Candida* score relative to max (per %)	0.06 (0.02–0.21)	<.001
COVID-19	13.33 (1.62–109.81)	.016
Hemato-oncologic malignancies	2.21 (1.18–4.15)	.014
Model 2		
Treatment consultation by an infectious diseases or microbiology consultant	0.32 (0.18–0.56)	<.001
Chronic liver disease	3.39 (1.48–7.80)	.004
Age (per year)	1.03 (1.01–1.04)	.005
Initial echinocandin treatment	0.45 (0.25–0.80)	.007
COVID-19	14.22 (1.71–118.06)	.014
Hemato-oncologic malignancies	2.01 (1.10–3.68)	.024

Abbreviations: CI, confidence interval; COVID-19, coronavirus disease 2019; HR, hazard ratio.

In the multivariable Cox regression models (Table 4), absence of treatment consultation by an infectious disease or clinical microbiology consultant, older age, hemato-oncologic malignancies, chronic liver disease, COVID-19, and the absence of initial treatment with an echinocandin, respectively, lower EQUAL *Candida* scores were independently associated with higher mortality.

## DISCUSSION

We performed a subanalysis of the multicenter, observational ECMM *Candida* III study, which included 632 candidemia patients from 64 hospitals across 20 European countries. We found that non-ICU patients had a higher burden of hemato-oncologic malignancies and higher CCI scores. Kaplan–Meier survival analysis confirmed a significantly lower survival probability (*P* < .001) in the ICU group. Multivariable analysis of ICU and non-ICU patients confirmed that ICU treatment and higher CCI were independent predictors of mortality. Within the cohort of non-ICU patients, postsurgical non-ICU patients had the highest survival rate and the longest hospital stay, while patients with hemato-oncologic malignancies had the lowest survival probability (*P* = .007). In the multivariable Cox regression analysis, the absence of treatment consultation by an infectious disease or clinical microbiology consultant, older age, hemato-oncologic malignancies, chronic liver disease, COVID-19, and initial treatment with another drug than an echinocandin, respectively, lower EQUAL *Candida* scores remained independently associated with mortality.

In addition, nutritional status appears to be an important factor, as malnutrition was a relevant predictor in non-ICU patients and has previously been associated with higher mortality [23]. Conversely, obesity may prolong the duration of infection and hospital stay but without consistently affecting mortality [24]. In contrast to previous reports describing non-ICU patients also as older [16, 17], our study showed no significant differences in age or sex distribution between ICU and non-ICU groups. Non-ICU patients underwent more ophthalmologic examinations (40.1% vs 24.2%; *P* < .001) and tended to receive more infectious disease consultations, although that finding was not statistically significant. As ocular candidiasis can be asymptomatic in early phases [25, 26], relying on patient-reported symptoms as a trigger for ophthalmologic examinations may prove unreliable and is naturally not possible in sedated ICU patients. Therefore, ICU patients who cannot report their symptoms are generally considered a prime population for receiving ophthalmoscopic screening.

Our findings show that stepdown to fluconazole occurred significantly more often in non-ICU patients than in ICU patients. Even after excluding patients who died within the first 14 days after diagnosis, non-ICU patients still underwent stepdown therapy more frequently than ICU patients.

In our cohort, *C. albicans* remained the most common fungal isolate in both ICU and in non-ICU patients. However, despite low absolute numbers, *P. kudriavzevii* was significantly more frequent in non-ICU, while *C. auris* was predominantly isolated from ICU patients. Similar results have been reported previously. For example, a post hoc analysis of the CANDIPOP project in Spain, which focused on candidemia acquired outside the ICU, identified *P. kudriavzevii* as 1 of the 5 most common isolated species after *C. albicans*, *C. parapsilosis*, *N. glabratus*, and *C. tropicalis* [16]. The SENTRY Antimicrobial Surveillance Program did not identify a significant difference in *P. kudriavzevii* prevalence between ICU and non-ICU cohorts [27]. In contrast, another study reported *P. kudriavzevii* more frequently in non-ICU patients compared with ICU patients (5.3% vs 2.2%) [28]. An Italian study also reported that *P. kudriavzevii* was associated with the highest mortality rate in both ICU and non-ICU patients, although not statistically significant [29]. In our cohort, 30-day mortality was significantly higher for non-ICU patients with *P. kudriavzevii* infection (66.3%) compared with ICU patients. Of note, this increased mortality cannot be attributed to an inadequate first-line therapy, as all patients with *P. kudriavzevii* infection received echinocandins as initial treatment, except 4 non-ICU patients (1.0%) who were treated with fluconazole. Notably, all 4 of these patients were alive at the last follow-up of the study. Furthermore, our observation of *C. auris* predominance in ICU patients is consistent with existing literature, which shows a rise in *C. auris* infections in ICUs, particularly during the COVID-19 pandemic [30, 31].

*Candida tropicalis* tended to be more prevalent in the hemato-oncologic malignancies group, consistent with previous reports. One study identified *C. tropicalis* as the most commonly isolated species in hematologic patients, and a study in pediatric hematologic malignancy patients reported *C. tropicalis* as the most frequently isolated species, accounting for 30% of cases [32]. The median duration of hospitalization among those who survived at least 30 days after diagnosis was significantly shorter in non-ICU patients versus ICU patients (21 days, IQR 10–35 days vs 27 days, IQR 14–49 days, *P* = .016). Postsurgical non-ICU patients had the longest hospitalization, likely reflecting the complexity of their underlying surgical conditions and the need for repeated interventions, rather than prolonged treatment of IC or candidemia. These patients were initially sufficiently stable to undergo surgical procedures and often required prolonged recovery due to IC [16, 33].

Among non-ICU patients, those with hemato-oncologic malignancies had the highest mortality (*P* < .001 in Kaplan–Meier analysis), while the lowest mortality was observed in patients post major surgery.

This finding is consistent with previous reports demonstrating higher mortality rates for candidemia in medical wards compared with surgical wards [16, 34]. One possible explanation is that surgical patients may have fewer chronic comorbidities, and their candidemia may be more related to perioperative factors rather than advanced underlying disease [35].

For the whole group of non-ICU patients, older age, hemato-oncologic malignancies, chronic liver disease, and COVID-19 remained significant independent baseline predictors of mortality. The COVID-19 pandemic in its early phase was not only associated with high mortality rates among those with acute respiratory failure in the ICU but also with an uptick in candidemia rates, triggered by overwhelmed and overcrowded ICUs [36]. Interestingly, our study found that COVID-19 was also a major factor associated with mortality risk in non-ICU patients, as was chronic liver disease, a known risk factor for candidemia and associated mortality, triggered by fungal translocation through the gut- and liver disease–associated immunocompromise [37]. In terms of management, consultation by an Infectious Diseases or Microbiology consultant, as shown previously [38], and higher EQUAL *Candida* scores reflecting guideline adherence in diagnosis and treatment, respectively, initial treatment with an echinocandin [21] remained strong independent predictors of survival.

The following limitations of our study should be noted. Data were not consistently available for all patients, and the results shown here reflect a realistic scenario without the use of standardized diagnostic strategies or treatment protocols for candidemia. Also, most participating centers were tertiary reference hospitals; therefore, results may be less applicable to district hospitals or other secondary care settings. Differences in access to diagnostics and antifungal treatment options in individual countries may limit the comparability of the results [39], and local diagnostic and treatment pathways may have influenced patient management and outcomes. While the sampling strategy, which included only the first ten consecutive candidemia patients per center and a limitation of centers per country depending on population size, ensured that the study sample was representative of candidemia across Europe, limitations may include underrepresentation of potential seasonal or outbreak-related variations. While the geographical distribution of the data reflects Europe and its overall laboratory capacity [6], centers with better access to diagnostics and antifungal therapies might be overrepresented. As this subanalysis is observational, residual confounding factors cannot be ruled out. While the number of cases per center and centers per country were restricted, we did not adjust for clustering by center or for differences in diagnostics and therapy availability between countries, which may have affected the accuracy of the *P*-values, HRs, and the interpretation of treatment patterns and outcomes. Another limitation was that the calculation of some other clinical scores, like the *Candida* score [40], was not possible as the necessary clinical data, for example, on colonization were not systematically collected, and that the Ostrosky-Zeichner score could not be evaluated for prediction of candidemia as no unmatched control group (ie, ICU patients without candidemia) was available. Finally, due to the small absolute number of *P. kudriavzevii* and *C. auris* cases some of the findings from subgroup analyses should be interpreted with caution.

## CONCLUSIONS

Patients with candidemia outside the ICU had more chronic comorbidities, including hematologic or oncologic diseases, but a higher survival rate than ICU patients, likely due to lower acute illness severity. While *C. albicans* was the predominant species in all groups and *C. auris* predominantly detected in the ICU, *P. kudriavzevii* was generally more common outside the ICU. Non-ICU patients more often underwent ophthalmoscopy, and postsurgical non-ICU patients had the highest survival rates and the longest hospital stays. In the non-ICU cohort, mortality was highest among hemato-oncologic malignancy patients.

Besides older age, hemato-oncologic malignancies, chronic liver disease, and COVID-19 were independently associated with mortality in non-ICU patients. In addition, several care-related factors were independently associated with mortality: the absence of consultation with an infectious disease or clinical microbiology specialist, the absence of initial treatment with an echinocandin, and lower EQUAL *Candida* scores. These findings could inform future guidelines and targeted interventions for non-ICU patients with candidemia, such as the implementation of systematic expert consultation or the emphasis on appropriate first-line antifungal therapy.

## Supplementary Material

ofag133_Supplementary_Data

## Supplementary Data

Supplementary materials are available at *Open Forum Infectious Diseases* online. Consisting of data provided by the authors to benefit the reader, the posted materials are not copyedited and are the sole responsibility of the authors, so questions or comments should be addressed to the corresponding author.
