# Supplementary material for: Clinical Characteristics, Management and Outcomes of Nonintensive Care Unit Candidemia: Subanalysis of the ECMM Candida III Multinational European Observational Cohort Study
Source: Open Forum Infect Dis. 2026 Mar 26;13(4):ofag133. doi: 10.1093/ofid/ofag133 (PMC13034534; doi:10.1093/ofid/ofag133)
Supplement: ofag133_Supplementary_Data [file ofag133_supplementary_data.docx]

**
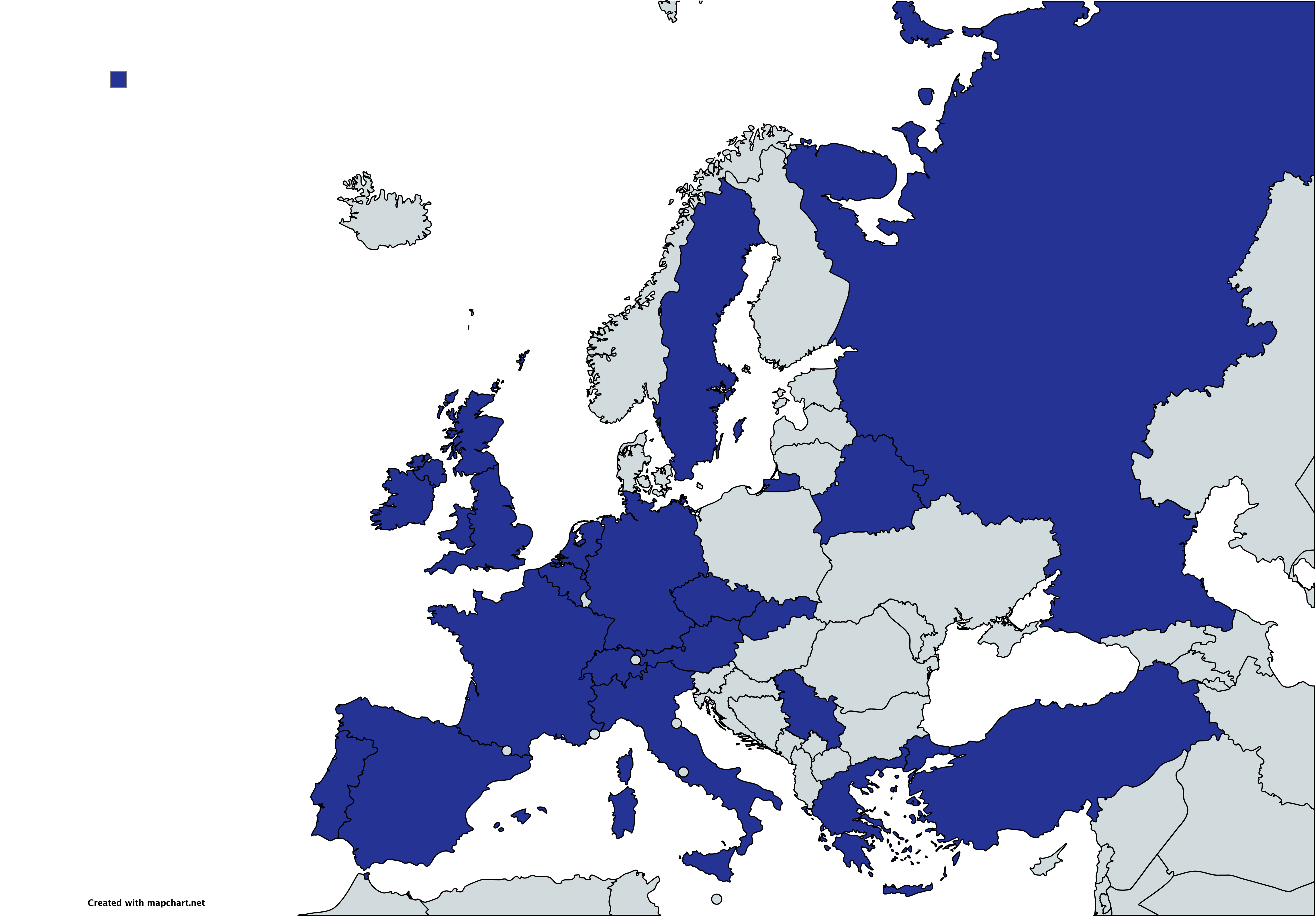
**

**Figure 1** Countries participating in the Candida III study (blue).

ALT TEXT

Map of Europe showing countries participating in the Candida III study shaded in dark blue, while non-participating countries are shown in light gray.

**Table 1** Number of non-ICU and ICU cases across participating countries.

|  | Study cohort (n=632) | non-ICU (n= 396) | ICU (n=236) | P value |
| --- | --- | --- | --- | --- |
| **Country** |  |  |  |  |
| Austria | 30 | 20 | 10 | <0.001 |
| Belarus | 2 | 2 | 0 |  |
| Belgium | 20 | 13 | 7 |  |
| Czech Republic | 23 | 11 | 12 |  |
| France | 73 | 59 | 14 |  |
| Germany | 73 | 23 | 50 |  |
| Greece | 18 | 11 | 7 |  |
| Ireland | 4 | 4 | 0 |  |
| Italy | 30 | 26 | 4 |  |
| Netherlands | 27 | 23 | 4 |  |
| Portugal | 10 | 5 | 5 |  |
| Russian Federation | 35 | 6 | 29 |  |
| Serbia | 29 | 28 | 1 |  |
| Slovakia | 10 | 10 | 0 |  |
| Slovenia | 10 | 8 | 2 |  |
| Spain | 47 | 39 | 8 |  |
| Sweden | 20 | 10 | 10 |  |
| Switzerland | 10 | 9 | 1 |  |
| Turkey | 66 | 38 | 28 |  |
| United Kingdom | 95 | 51 | 41 |  |

**Table 2** Distribution of Candida species among non-ICU and ICU patients in participating countries.

|  | ***C. albicans*** | | ***N. glabratus*** | | ***C. parapsilosis*** | | ***C. tropicalis*** | | ***P. kudriavzevii*** | | ***C. auris*** | | ***C. dubliniensis*** | | ***C. lusitaniae*** | | ***K. marxianus*** | | ***M. guilliermondii*** | | ***Others*** | | **unidentified** | |
| --- | --- | --- | --- | --- | --- | --- | --- | --- | --- | --- | --- | --- | --- | --- | --- | --- | --- | --- | --- | --- | --- | --- | --- | --- |
|  | **non-ICU** | **ICU** | **non-ICU** | **ICU** | **non-ICU** | **ICU** | **non-ICU** | **ICU** | **non-ICU** | **ICU** | **non-ICU** | **ICU** | **non-ICU** | **ICU** | **non-ICU** | **ICU** | **non-ICU** | **ICU** | **non-ICU** | **ICU** | **non-ICU** | **ICU** | **non-ICU** | **ICU** |
| **Austria** | 9 | 3 | 0 | 1 | 1 | 0 | 0 | 1 | 1 | 0 | 0 | 0 | 0 | 1 | 0 | 0 | 0 | 0 | 0 | 0 | 1 | 0 | 0 | 0 |
| **Belarus** | 1 | 0 | 0 | 0 | 0 | 0 | 0 | 0 | 1 | 0 | 0 | 0 | 0 | 0 | 0 | 0 | 0 | 0 | 0 | 0 | 0 | 0 | 0 | 0 |
| **Belgium** | 7 | 2 | 5 | 1 | 0 | 0 | 1 | 1 | 0 | 0 | 0 | 0 | 0 | 0 | 0 | 0 | 0 | 0 | 0 | 0 | 0 | 0 | 0 | 0 |
| **Czech Republic** | 8 | 4 | 1 | 5 | 0 | 1 | 1 | 0 | 1 | 0 | 0 | 0 | 0 | 1 | 0 | 0 | 0 | 0 | 0 | 1 | 0 | 0 | 0 | 0 |
| **France** | 24 | 5 | 16 | 4 | 6 | 1 | 6 | 3 | 2 | 0 | 0 | 0 | 0 | 0 | 3 | 0 | 2 | 0 | 0 | 1 | 2 | 1 | 0 | 0 |
| **Germany** | 12 | 28 | 8 | 12 | 0 | 3 | 1 | 5 | 0 | 0 | 0 | 0 | 1 | 2 | 0 | 0 | 1 | 1 | 0 | 0 | 0 | 0 | 0 | 0 |
| **Greece** | 6 | 1 | 0 | 0 | 2 | 6 | 1 | 0 | 1 | 0 | 0 | 0 | 0 | 0 | 0 | 0 | 0 | 0 | 0 | 0 | 1 | 0 | 0 | 0 |
| **Ireland** | 2 | 0 | 1 | 0 | 0 | 0 | 1 | 0 | 0 | 0 | 0 | 0 | 0 | 0 | 0 | 0 | 0 | 0 | 0 | 0 | 0 | 0 | 0 | 0 |
| **Italy** | 11 | 2 | 3 | 0 | 5 | 2 | 2 | 0 | 0 | 0 | 0 | 0 | 0 | 0 | 0 | 0 | 0 | 0 | 0 | 0 | 0 | 0 | 0 | 0 |
| **Netherlands** | 13 | 3 | 3 | 1 | 6 | 0 | 1 | 0 | 0 | 0 | 0 | 0 | 0 | 0 | 0 | 0 | 0 | 0 | 0 | 0 | 0 | 0 | 0 | 0 |
| **Portugal** | 1 | 3 | 2 | 1 | 1 | 1 | 0 | 0 | 0 | 0 | 0 | 0 | 0 | 0 | 1 | 0 | 0 | 0 | 0 | 0 | 0 | 0 | 0 | 0 |
| **Russian Federation** | 4 | 7 | 1 | 2 | 0 | 1 | 0 | 1 | 0 | 0 | 1 | 12 | 0 | 0 | 0 | 0 | 0 | 0 | 0 | 2 | 0 | 0 | 0 | 5 |
| **Serbia** | 19 | 0 | 4 | 1 | 1 | 0 | 3 | 0 | 2 | 0 | 0 | 0 | 0 | 0 | 0 | 0 | 0 | 0 | 0 | 0 | 0 | 0 | 0 | 0 |
| **Slovakia** | 4 | 0 | 3 | 0 | 1 | 0 | 1 | 0 | 0 | 0 | 0 | 0 | 0 | 0 | 1 | 0 | 0 | 0 | 0 | 0 | 0 | 0 | 0 | 0 |
| **Slovenia** | 4 | 0 | 6 | 0 | 0 | 0 | 0 | 0 | 0 | 0 | 0 | 0 | 0 | 0 | 0 | 0 | 0 | 0 | 0 | 1 | 0 | 0 | 0 | 0 |
| **Spain** | 14 | 2 | 6 | 3 | 11 | 1 | 4 | 0 | 1 | 1 | 0 | 0 | 0 | 0 | 0 | 0 | 0 | 0 | 0 | 1 | 1 | 0 | 0 | 0 |
| **Sweden** | 3 | 37 | 5 | 2 | 2 | 0 | 1 | 0 | 0 | 0 | 0 | 0 | 0 | 1 | 0 | 0 | 0 | 0 | 0 | 0 | 0 | 0 | 0 | 0 |
| **Switzerland** | 5 | 0 | 2 | 1 | 0 | 0 | 1 | 0 | 0 | 0 | 0 | 0 | 1 | 0 | 0 | 0 | 0 | 0 | 0 | 0 | 0 | 0 | 0 | 0 |
| **Turkey** | 13 | 12 | 6 | 3 | 3 | 11 | 6 | 1 | 3 | 0 | 0 | 1 | 0 | 0 | 1 | 0 | 0 | 1 | 0 | 0 | 3 | 0 | 1 | 0 |
| **United Kingdom** | 15 | 26 | 15 | 9 | 13 | 4 | 3 | 1 | 2 | 1 | 0 | 1 | 2 | 1 | 0 | 0 | 0 | 0 | 0 | 0 | 1 | 0 | 0 | 0 |

**Table 3** Univariable and multivariable Cox regression model for predictors of mortality for non-ICU and ICU patients

|  | **Univariable HR (95% CI)** | **P value** |
| --- | --- | --- |
| **Treatment in ICU** | 1.76 (1.39-2.23) | <0.001 |
|  |  |  |
| **Sex (female vs. male)** | 1.19 (0.93-1.51) | 0.167 |
|  |  |  |
| **Age at candidemia diagnosis** |  |  |
| 18-29 years | 1 (ref.) |  |
| 30-49 years | 2.05 (0.63-6.66) | 0.232 |
| 50-69 years | 2.77 (0.88-8.69) | 0.081 |
| 70-89 years | 3.76 (1.19-11.85) | 0.024 |
| > 90 years | 4.33 (1.19-15.74) | 0.026 |
|  |  |  |
| **Underlying diseases** |  |  |
| Haematological/Oncological malignancy |  |  |
| Leukaemia | 1 (ref.) |  |
| Lymphoma | 1.36 (0.72-2.55) | 0.344 |
| Multiple Myeloma | 0.98 (0.42-2.3) | 0.961 |
| Solid tumour | 1.04 (0.64-1.71) | 0.872 |
| Other | 0.78 (0.33-1.83) | 0.566 |
|  |  |  |
| Rheumatic disease/Autoimmune disorder | 1.43 (0.9-2.25) | 0.127 |
| Chronic cardiovascular disease | 1.32 (1.02-1.72) | 0.036 |
| Chronic liver disease | 1.51 (1.06-2.15) | 0.024 |
| Chronic pulmonary disease | 1.3 (0.88-1.91) | 0.186 |
| Acute or chronic renal disease | 1.23 (0.94-1.62) | 0.139 |
| Diabetes mellitus | 1 (0.76-1.32) | 1.000 |
| HIV/AIDS | 0.79 (0.25-2.46) | 0.681 |
| Coronavirus disease 2019 (COVID-19) | 1.43 (0.82-2.51) | 0.432 |
|  |  |  |
| **Risk factors for candidemia** |  |  |
| Solid organ transplantation | 0.72 (0.32-1.62) | 0.432 |
| Other disorders requiring or causing immunosuppression | 1.1 (0.65-1.84) | 0.118 |
| Charlson Comorbidity Index (CCI) | 1.09 (1.05-1.13) | <0.001 |
| Major surgery (not including surgery as antifungal therapy) | 0.94 (0.72-1.24) | 0.661 |
| Other risk factors (e.g. prosthetic material e.g. prosthetic valve/foreign body) | 0.95 (0.67-1.34) | 0.761 |
| Trauma | 1.2 (0.67-2.14) | 0.536 |
| Alcoholism | 1.22 (0.8-1.88) | 0.537 |
| IV drug abuse | 0.57 (0.24-1.38) | 0.231 |
| Low albumin level | 1.45 (1.15-1.84) | 0.002 |
| Obesity (BMI > 30) | 0.95 (0.64-1.4) | 0.794 |
| Central venous catheter (CVC) | 1.38 (1.07-1.79) | 0.015 |
| Extracorporeal membrane oxygenation (ECMO) | 1.3 (0.64-2.62) | 0.468 |
| Total parenteral nutrition | 0.85 (0.64-1.14) | 0.28 |
|  |  |  |
| **Consultation** |  |  |
| Infectious disease consultant | 1(ref.) |  |
| Microbiology consultant | 0.95 (0.71-1.26) | 0.7 |
| No consultation | 1.58 (1.17-2.13) | 0.003 |
|  |  |  |
| **Diagnostics** |  |  |
| Initial blood cultures (40 mL) | 0.66 (0.44-0.93) | 0.025 |
| Species identification | 0.58 (0.32-0.87) | 0.009 |
| Susceptibility testing | 0.50 (0.36-0.68) | <0.001 |
| Echocardiography | 0.42 (0.33-0.53) | <0.001 |
| Ophthalmoscopy | 0.26 (0.20-0.35) | <0.001 |
|  |  |  |
| **Management** |  |  |
| Initial echinocandin treatment | 0.56 (0.46-0.73) | <0.001 |
| Stepdown to fluconazole | 0.38 (0.28-0.53) | <0.001 |
|  |  |  |
|  | **Multivariable HR (95% CI)** | **P value** |
|  |  |  |
| Treatment in ICU | 1.90 (1.49-2.41) | <0.001 |
| Charlson Comorbidity Index (CCI) | 1.10 (1.06-1.14) | <0.001 |
| Sex | 1.14 (0.91-1.47) | 0.242 |
| Age at Diagnosis | 1.17 (0.98-1.41) | 0.062 |

COVID-19 = Coronavirus disease 2019, IQR = interquartile range, BMI = body mass index, CVC = Central venous catheter,

ECMO = Extracorporeal membrane oxygenation, CCI = Charlson Comorbidity Index
